# Biosynthesis Strategies and Application Progress of Mandelic Acid Based on Biomechanical Properties

**DOI:** 10.3390/microorganisms13081722

**Published:** 2025-07-23

**Authors:** Jingxin Yin, Yi An, Haijun Gao

**Affiliations:** School of Life Science, Beijing Institute of Technology, Beijing 100081, China; yinjingxin04@163.com (J.Y.); anyi@bit.edu.cn (Y.A.)

**Keywords:** mandelic acid, biomechanics, biosynthesis, microbial cell

## Abstract

Mandelic acid (MA), as an important chiral aromatic hydroxy acid, is widely used in medicine, the chemical industry, and agriculture. With the continuous growth of market demand, traditional chemical synthesis methods are increasingly inadequate to meet the requirements of green and sustainable development due to issues such as complex processes, poor stereoselectivity, numerous byproducts, and serious environmental pollution. MA synthesis strategies based on biocatalytic technology have become a research hotspot due to their high efficiency, environmental friendliness, and excellent stereoselectivity. Significant progress has been made in enzyme engineering modifications, metabolic pathway design, and process optimization. Importantly, biomechanical research provides a transformative perspective for this field. By analyzing the mechanical response characteristics of microbial cells in bioreactors, biomechanics facilitates the regulation of relevant environmental factors during the fermentation process, thereby improving synthesis efficiency. Molecular dynamics simulations are also employed to uncover stability differences in enzyme–substrate complexes, providing a structural mechanics basis for the rational design of highly catalytically active enzyme variants. These biomechanic-driven approaches lay the foundation for the future development of intelligent, responsive biosynthesis systems. The deep integration of biomechanics and synthetic biology is reshaping the process paradigm of green MA manufacturing. This review will provide a comprehensive summary of the applications of MA and recent advances in its biosynthesis, with a particular focus on the pivotal role of biomechanical characteristics.

## 1. Introduction

**Figure 1:**
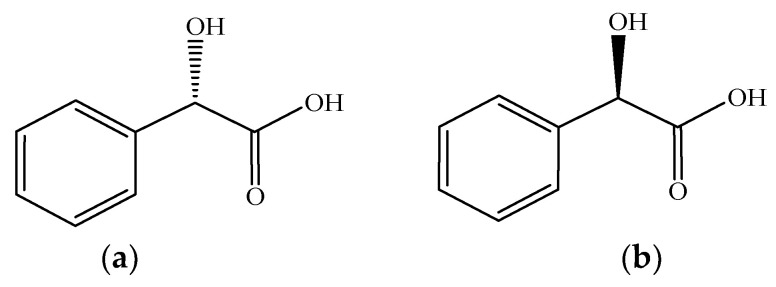
Chemical structures of mandelic acid (MA). (**a**) Chemical structures of (S)-MA; (**b**) Chemical structures of (R)-MA.

Mandelic acid (MA), also known as α-hydroxyphenylacetic acid or amygdalic acid, is a fine chemical with a chiral center [1,2]. Its enantiomers (Figure 1) have garnered significant attention due to their distinct pharmacodynamic profiles. Single-enantiomer products not only exhibit higher bioavailability but also significantly reduce the incidence of adverse reactions in pharmaceutical formulations. As a key intermediate in chiral drug development, MA plays a vital role in the synthesis of antibiotics (e.g., penicillins and cephalosporins), anticancer agents, antithrombotic agents, and anti-inflammatory drugs [2,3]. It also serves as a chiral resolving agent in nuclear magnetic resonance (NMR) analysis and chromatographic separation techniques [4,5,6], demonstrating broad application prospects in the pharmaceutical, chemical, and agricultural industries [7,8,9].

However, traditional chemical synthesis of MA faces challenges such as limited product purity and high costs associated with chiral resolution. In contrast, biosynthetic technologies harness engineered enzyme systems and microbial cell factories to efficiently produce optically pure MA from renewable substrates, such as glucose and glycerol, through enzyme cascade reactions.

Notably, the integration of biomechanics research is injecting transformative momentum into this technological framework and opening new dimensions for optimization. At the bioreactor scale, precise regulation of fluid mechanical conditions within bioreactors [10] enables tuning of the cellular mechanical environment to enhance mass transfer dynamics across cell membranes, ultimately improving substrate conversion rates. On the molecular scale, molecular biomechanical studies, particularly through molecular dynamics simulations, reveal how the hydrogen bond network and van der Waals force field within enzyme active sites undergo dynamic mechanical responses (such as elastic deformation energy storage and release). These responses critically regulate substrate binding affinity, transition-stabilization, and catalytic efficiency toward stereoselective reactions [11,12]. For instance, studies suggest nitrilase active pockets may utilize conformational strain to release mechanical energy, thereby driving stereoselective catalysis [11,12]. At the cellular scale, the perspective of cellular biomechanics further demonstrates that the efficiency of substrate transmembrane transport in microbial factories is governed by key mechanical parameters, including cell membrane tension and osmotic pressure gradients. Importantly, force-induced conformational changes in membrane proteins (e.g., transporters) can actively reshape the distribution of metabolic flux within the cell [13,14].

Molecular dynamics simulations are employed not only to analyze substrate deformation [15] but, more significantly, to elucidate the mechanical stability and dynamic response of enzyme–substrate complexes. This deeper understanding facilitates the rational construction of stereoselective engineered enzymes and provides a crucial theoretical foundation for the directional synthesis of target enantiomers. This review systematically summarizes recent advances in MA applications across pharmaceuticals, chemical engineering, and agriculture. It focuses on analyzing biosynthetic strategies for both (S)-MA and (R)-MA from the perspective of substrate metabolic pathways. Critically, it explores and highlights innovative applications of biomechanical approaches in metabolic network reconstruction and enzymatic catalysis regulation, offering a unique and powerful lens to advance the field. These insights aim to provide theoretical references for the green manufacturing of chiral pharmaceuticals.

## 2. Methodology

### 2.1. Literature Search and Screening Strategy

This study adopted a systematic literature review method, aiming to comprehensively integrate the multidisciplinary research progress related to MA biosynthesis and its biomechanical properties. The literature search was carried out based on the following strategy.

#### 2.1.1. Database and Search Scope

PubMed, Web of Science, ScienceDirect and SpringerLink are the core databases, supplemented by professional chemical and bioengineering journals (such as Applied Microbiology and Biotechnology, Journal of Agricultural and Food Chemistry). The search time span is from 2000 to 2025, focusing on high-impact research in the past six years (2019–2025).

#### 2.1.2. Keyword Combination

The main search terms include “mandelic acid biosynthesis”, “biomechanics in biocatalysis”, “enzyme engineering”, “microbial cell factories”, “stereoselective synthesis”, and are extended to related terms (such as “nitrilase modification”, “metabolic flux analysis”, “fluid shear stress”). Boolean logic operators (AND/OR) are used to construct compound search formulas, for example: (mandelic acid) AND (biomechanics OR enzyme dynamics) AND (synthesis OR metabolic pathway).

#### 2.1.3. Screening Criteria

After the initial search, the titles and abstracts were used to screen out literature directly related to the mechanism of MA synthesis, biomechanical regulation, and industrial application. Inclusion criteria included: (1) experimental or computational studies; (2) clear involvement of MA synthesis pathways or biomechanical mechanisms; (3) provision of reproducible data or method details. Exclusion criteria included: (1) non-English literature; (2) non-peer-reviewed content (such as conference abstracts); (3) repetitive studies or lack of innovative conclusions.

After the initial search, the titles and abstracts were screened to identify literature directly related to the mechanisms of MA synthesis, biocatalytic regulation, and industrial applications. The inclusion criteria were as follows: (1) experimental or computational studies; (2) clear focus on MA synthesis pathways or biocatalytic mechanisms; and (3) provision of reproducible data or detailed methodologies. The exclusion criteria included: (1) non-English publications; (2) non-peer-reviewed materials (e.g., conference abstracts); and (3) duplicate studies or those lacking novel conclusions.

### 2.2. Literature Analysis and Integration Framework

#### 2.2.1. Subject Classification and Coding

The selected literature is divided into four categories according to the research topic: (a) chemical and biological synthesis methods; (b) enzyme engineering and molecular dynamics simulation; (c) microbial cell factory optimization; (d) multi-field application of MA. Each type of literature is further coded into sub-topics (such as “nitrilase directed evolution” and “membrane tension regulation”), and an Excel database is established to record the core parameters of the literature (such as enzyme activity, product enantiomeric excess, fermentation efficiency).

The selected literature was categorized into four main research areas: (a) chemical and biological synthesis methods; (b) enzyme engineering and molecular dynamics simulations; (c) optimization of microbial cell factories; and (d) multidisciplinary applications of MA. Each category was further coded into subtopics (e.g., “nitrilase directed evolution” and “membrane tension regulation”). An Excel database was created to record key parameters from each study, such as enzyme activity, product enantiomeric excess, and fermentation efficiency.

#### 2.2.2. Quality Assessment and Data Extraction

A hierarchical quality assessment approach was adopted. First, high-credibility literature was selected based on journal impact factor (IF ≥ 2.0) and citation frequency (≥5 times). Next, the rigor of the experimental design was evaluated, including the presence of control groups and assessment of statistical significance. Data extraction focused on synthesis efficiency (e.g., conversion rate and yield), stereoselectivity (ee value), and biomechanical parameters such as shear stress threshold and intermolecular force strength.

#### 2.2.3. Review Structure Design

The literature is organized according to the logical framework of “synthesis strategy–application progress–technical challenges.” The synthesis section is further subdivided by substrate type (e.g., mandelonitrile, mandelate), with an emphasis on the influence of biomechanical mechanisms on catalytic efficiency. The application section is classified into medicine, chemical industry, and agriculture, and incorporates representative case studies to analyze the relationship between biomechanical properties and functional performance. The discussion of technical challenges and future directions is based on recurring bottlenecks identified in the literature—such as limited transmembrane transport efficiency and imbalances in multi-enzyme synergy—for which systematic solutions are proposed.

### 2.3. Limitations

This study may have the following limitations: (1) the potential omission of non-English literature; (2) limited data coverage for certain emerging technologies (such as AI-assisted enzyme design) due to their recent emergence; and (3) a lack of industrial-scale data, with most research remaining at the laboratory scale. Future research should incorporate patent databases and industry reports to improve the comprehensiveness of the technology transfer analysis.

## 3. The Application of MA

MA is experiencing rapid global market expansion due to its multifaceted applications in pharmaceuticals, cosmetics, chemicals, textiles, and agriculture [3,4,5,6,9]. According to a research report by Spherical Insights and Consulting, the global mandelic acid market was valued at USD 190 million in 2021 [https://www.sphericalinsights.com/reports/mandelic-acid-market (accessed on 8 July 2025)], and is expected to grow significantly to USD 955.03 million by 2032, with a compound annual growth rate (CAGR) of 14.42% during the forecast period [https://www.credenceresearch.com/report/mandelic-acid-market (accessed on 8 July 2025)]. From a regional perspective, North America is expected to maintain a major market share due to its advanced cosmetics industry, while Asia-Pacific is forecasted to achieve the fastest growth, particularly in countries such as China, South Korea, and Japan. Europe continues to expand its market due to rising consumer preference for organic and natural skincare solutions [https://www.marketresearchfuture.com/reports/mandelic-acid-market-20300 (accessed on 8 July 2025)].

### 3.1. Pharmaceuticals Field

In the medical field, the biomechanical properties of MA and its derivatives are increasingly driving the development of new therapeutic strategies (Table 1). Studies have shown that by specifically binding to the V3 loop region of the HIV-1 viral surface protein, the glycolic acid condensate SAMMA can significantly reduce the interaction force between the virus and the host cell membrane [16,17,18]. This innovative biomechanical intervention increases the critical curvature radius threshold required for viral fusion, thereby effectively blocking the membrane fusion process [19]. Atomic force microscopy observations further confirmed that SAMMA treatment directly weakens the driving force of the *Chlamydia* flagellar motor, thereby causing it to lose its ability to invade tissue [16,20]. In dermatology, MA not only inhibits tyrosinase activity to improve pigmentation in photoaged skin but also accelerates post-laser epidermal repair by remodeling the viscoelastic properties of epidermal tissue. It also regulates keratinocyte differentiation for the treatment of inflammatory acne [21,22,23,24].

Enantiomeric derivatives of MA exhibit unique advantages in precision medicine. (R)-O-chloroglycolic acid is a key chiral precursor of the antithrombotic drug clopidogrel. It irreversibly inhibits the platelet P2Y12 receptor by altering its three-dimensional conformation and enhancing the steric hindrance of the ADP binding site [25,26]. (S)-MA is primarily used in synthesizing urological and anti-inflammatory drugs, serving as a critical precursor for (S)-oxybutynin, a medication that reduces bladder and urinary tract muscle spasms in patients with overactive bladder syndrome [27]. In anti-inflammatory drug design, molecular dynamics simulations have optimized the binding free energy distribution of the (S)-glycolic acid–COX-2 complex. (S)-glycolic acid forms a highly stable complex with the COX-2 enzyme by refining the binding energy profile [28]. This complex can maintain conformational stability under physiological shear stress [29]. Based on this mechanical adaptation mechanism, selective inhibitors such as celecoxib (Celebrex^®^) and deracoxib have been developed, whose enhanced mechanical stability significantly reduces the rate of drug dissociation [3].

Driven by rising demand in the pharmaceutical and personal care sectors, MA’s market expansion is largely anchored in its medical efficacy. Notably, biomechanical research is enabling functional improvements in MA-derived drugs. Trimethylcyclohexyl mandelate (a microcirculation enhancer) improves microvascular flow by modulating erythrocyte membrane shear deformability, with efficacy directly linked to its regulation of hemodynamic shear stress. The optimized bactericidal efficiency of methenamine mandelate (a urinary antiseptic) benefits from a sustained-release formulation design. In antibiotic development, molecular docking simulations of cephalosporin–target protein complexes allow prediction of the relationship between mechanical stability and the antibacterial activity of β-lactam rings, providing critical insights for next-generation drug design [30,31,32].

**Table 1:** Application of mandelic acid (MA) in Pharmaceuticals field.

Domain	Category	Core Function	Typical Applications	References
Pharmaceuticals	Antiviral Drugs	Block viral transmission and membrane fusion	SAMMA (inhibits HIV dendritic cell transmission, prevents HSV infection)	[16,17,18]
	Dermatological Agents	Regulate pigmentation and epidermal repair	Creams for photoaging/acne treatment; post-laser repair gels	[21,22,23,24]
	Cardiovascular Drugs	Inhibit platelet aggregation	(R)-O-Chloromandelic acid Clopidogrel (P2Y12 receptor antagonist); Trimethylcyclohexyl mandelate (microcirculation enhancer)	[2,25,26]
	Urological/Anti-inflammatory Drugs	Modulate bladder smooth muscle and inflammatory mediators	(S)-Oxybutynin (overactive bladder treatment); Methenamine mandelate (urinary antiseptic); Celecoxib and Deracoxib (COX-2 inhibitors)	[3,27,28,29]
	Antibiotic Synthesis	Disrupt bacterial cell wall synthesis	Cephalosporin antibiotic sidechain construction	[30,31,32]

### 3.2. Chemical Industry

In the chemical industry, MA exhibits the dual advantages of unique chirality and high molecular modifiability (Table 2). As a classical chiral auxiliary [5,6], its stereochemical recognition sites—formed by the benzene ring and hydroxyl group—play a pivotal role in supramolecular recognition. MA can not only create chiral-selective environments through hydrogen-bond networks to enable efficient chromatographic separation of chiral alcohols, but also serve as a functional monomer in molecularly imprinted materials, facilitating kinetic resolution of (S)-enantiomers through differences in intermolecular interactions [33,34]. Optically pure MA further extends its utility as a multifunctional analytical reagent. The ortho-hydroxyl group can form stable chelates with zirconium ions, making it a sensitive indicator for trace zirconium detection, while its carboxyl group can undergo condensation reactions with ketones, offering novel strategies for developing spectrophotometric methods to analyze ketone compounds [35].

In fine chemical synthesis, MA-derived benzodifuranone-based disperse dyes demonstrate excellent color fastness and vivid hues due to their planar conjugated structures. These dyes are especially suitable for low-temperature dyeing processes of polyester microfibers, effectively overcoming the migration issues often seen with traditional dyes on microfiber surfaces [36]. Poly mandelic acid (PMA), a biodegradable polymer, shows promise as a potential substitute for styrene-based plastics in packaging applications [37]. In addition, its synthetic intermediate, mandelonitrile, plays a critical role in molecular transformations. Through versatile functional group conversions, mandelonitrile enables the synthesis of a diverse range of compounds: hydrolysis or oxidation yields hydroxyphenyl glyoxylic acid derivatives, while degradation produces phenylglycine-type antibiotic side chains. These transformations are indispensable in the synthesis of surfactants, liquid crystal materials, and β-lactam antibiotics [38].

This molecular transformation versatility reinforces MA’s status as a key hub in the fine chemical industry. With the global MA market expanding, demand from the chemical and textile sectors is expected to significantly drive overall growth, particularly in fine chemical synthesis and biodegradable polymer development.

**Table 2:** Application of MA in Chemical Industry.

Domain	Category	Core Function	Typical Applications	References
Chemical Industry	Chiral Separation Materials	Construct supramolecular recognition systems	Chiral stationary phases for chromatography; molecularly imprinted resolution materials	[5,6,33,34]
	Analytical Reagents	Specific metal chelation and condensation reactions	Zirconium ion detection reagents; ketone spectrophotometric probe	[35]
	Advanced Dye Synthesis	Enhance fiber dyeing performance	Benzodifuranone-based disperse dyes	[36]
	Eco-friendly Materials	Synthesize biodegradable plastics	Poly mandelic acid (PMA) as a biodegradable polymer	[37]
	Fine Chemical Intermediates	Multifunctional group conversion platform	α-Aminonitriles, phenylglyoxylic acid, phenylglycine derivatives	[38]

### 3.3. Agricultural Field

In agricultural plant protection, MA has successfully transitioned from being a natural product to high-efficiency agrochemicals through structural modifications [9,39]. Its molecular framework, subjected to targeted modifications such as halogenation and esterification, allows for precise targeting of diverse agricultural pathogens (Table 3). For oomycete diseases (e.g., tomato late blight, potato late blight), MA derivatives significantly reduce the sporangium germination rates of *Phytophthora infestans* by interfering with cell membrane sterol synthesis and altering the mechanical properties of the lipid bilayer [40,41]. The molecular mechanical properties of glycolic acid derivatives are driving the rational design of new pesticides. Maniphrid is anchored to the pathogen cell membrane through the hydrophobic interaction of its diacetylene group. This mechanical locking effect enables the glycolic acid group to precisely target the catalytic domain of phosphatidylinositol synthase [42], taking advantage of the molecular dynamics that optimize the binding site’s mechanical stability. The compound exerts torque to hinder the conformational flipping of the ion pairs of the amino acid residues in the enzyme’s active center, thereby reducing its catalytic efficiency. Compared with benzamide fungicides, this dual mechanism greatly improves efficacy against oomycete diseases [43,44]. Experiments have shown that the critical expansion pressure required for the germination of *Phytophthora* sporangia significantly increased after treatment, effectively inhibiting disease development [45].

For ascomycete pathogens, new glycolate fungicides achieve precise control by regulating the distribution of mechanical stress at the top of the hyphae. For ascomycete diseases such as grape downy mildew, pathogens are controlled by inhibiting the secretion of growth proteins at the top of the fungal hyphae [43,44]. Real-time microscopic imaging intuitively shows that after the compound acts, the calcium ion gradient at the top of the hyphae slows down, and the tension distribution of the cytoskeleton becomes disordered. This is similar to dismantling the “building scaffolding” of the pathogen, making it unable to form the cell wall normally [46].

In terms of insecticide innovation, pyrethroid analogs synthesized with MA as the chiral source show unique spatial stress regulation capabilities. The α-cyano group in its molecular structure forms a cis configuration match with the pyrethroid ester group, which is inserted into the gate structure of the pest sodium ion channel as precisely as a customized key, and the blocking efficiency is several times higher than that of traditional insecticides [47]. Additionally, the MA-derived herbicide metamitron suppresses photosynthesis by modulating the mechanical response threshold of plant stomatal opening/closing, effectively controlling both gramineous and broadleaf weeds [48]. Although agriculture represents a relatively smaller share of the global MA market, the demand for MA-based agrochemicals is expected to grow due to rising interest in low-resistance and biomechanically optimized pesticides.

**Table 3:** Application of MA in Agriculture field.

Domain	Category	Core Function	Typical Applications	References
Agriculture	Fungicides	Disrupt pathogen membrane structure and metabolism	Mandipropamid (phosphatidylinositol synthase inhibitor); downy mildew control agents; ascomycota pathogen control	[43,44,46]
	Insecticides	Block insect neural signaling	Cypermethrin analogs (sodium channel modulators)	[47]
	Herbicides	Inhibit photosynthetic systems	Metamitron (photosynthesis inhibitor)	[48]

## 4. Synthetic Methods of MA

### 4.1. Chemical Synthesis Method

The chemical synthesis pathways of MA can be primarily categorized into traditional chemical synthesis methods and novel catalytic synthesis technologies. Among traditional approaches, the benzaldehyde cyanohydrin hydrolysis method and phenacyl halide hydrolysis method have been most extensively applied [49]. The former involves hydrogen cyanide addition to benzaldehyde to form a cyanohydrin intermediate, followed by acidic hydrolysis to yield MA. The latter utilizes halogen substitution at the α-position of acetophenone followed by hydrolysis to prepare the product [50,51]. Although these methods are based on mature technologies and offer wide applicability, they suffer from several critical drawbacks. These conventional processes require high-temperature and high-pressure conditions, employ highly toxic reagents such as cyanides or halogenated compounds, and typically rely on precious metal catalysts. Such requirements lead to increased energy consumption and operational risks. Moreover, these processes generate substantial amounts of hazardous cyanide- and halogen-containing wastewater, posing serious environmental risks and increasing downstream treatment burdens. Therefore, despite their industrial maturity, these methods are increasingly scrutinized due to their environmental impact, safety hazards, and sustainability concerns.

To address these traditional synthesis bottlenecks, several improved processes have been subsequently developed:

(1) Phase-transfer catalysis employs quaternary ammonium salt catalysts to enable efficient synthesis under ambient conditions by enhancing mass transfer in biphasic systems, while avoiding the use of strong oxidizing or reducing agents [52]. This method benefits from mild reaction conditions and operational simplicity. However, it also faces several technical drawbacks, such as catalyst leaching, poor catalyst recyclability, and extended reaction times, which limit its industrial feasibility.
(2) Asymmetric synthesis utilizes chiral ligands to directly produce enantiomerically pure (R)-MA or (S)-MA with high optical purity [53]. This approach provides excellent enantiomeric excess (ee > 98%), making it attractive for high-value applications. Nevertheless, its practical use is hindered by the high cost of chiral catalysts and the need for inert atmosphere equipment, which substantially raises production expenses and operational complexity.
(3) Optical resolution methods combine chemical synthesis with chiral separation techniques to isolate optically pure products [54]. Although these methods are capable of obtaining enantiopure compounds, they generally exhibit low separation efficiency, difficulties in recovering resolving agents, and low overall yield in industrial settings. As a result, optical resolution remains challenging for large-scale or economically viable application.

A comparative summary of these chemical synthesis approaches, highlighting their respective advantages and limitations, is presented in Table 4.

### 4.2. Biosynthesis Method

**Figure 2:**
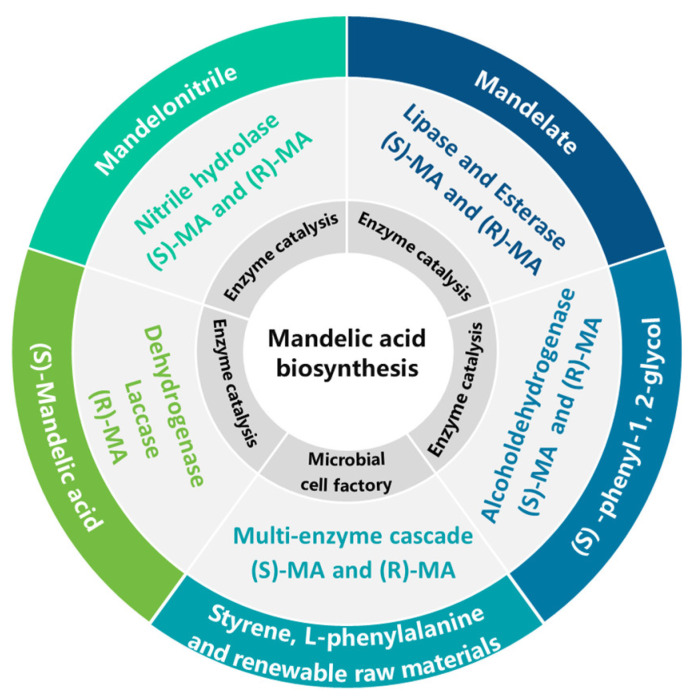
MA biosynthesis with different substrates.

The biosynthesis of MA leverages the high efficiency and stereoselectivity of enzyme catalytic systems, with biomechanical studies providing novel perspectives for enzyme molecular design and catalytic process optimization. Current research primarily focuses on four major classes of enzymes: nitrilases [55,56], lipases and esterases [57,58], dehydrogenases, and laccases [59,60]. The main features and limitations of these biosynthesis approaches are summarized in Table 5, with the schematic overview of MA biosynthesis from different substrates depicted in Figure 2.

#### 4.2.1. Rational Design of Nitrilase Catalytic Systems

The stereoselectivity of nitrilases (EC 3.5.5.1) originates from the mechanical constraint effects of their active-site pockets. Like a precise molecular mold, the cysteine at the active site forms a hydrogen bond network through the thioamide intermediate. This directional difference in molecular tension determines the spatial orientation of the product [55,56]. In the design of catalytic systems, researchers have found that the mechanical properties of key residues play a decisive role in the formation of substrate transition states [61]. Sun et al. used molecular dynamics simulation to reveal that the M113 residue of the psychrophilic *Pseudomonas* PpL19 nitrilase produces a nanoscale radial stress field during catalysis [62]. This mechanical stimulation causes a slight deformation of the triple bond of the mandelonitrile, significantly reducing the activation energy of the reaction (4.6 kcal/mol). The M113F mutation optimizes the substrate orientation angle to 15° through the π–π stacking effect between the benzene rings, just like adding a positioning device to the molecule, so that the enantiomeric excess (ee) reaches 98% [62]. Using QM/MM simulation, the Bian group found that the guanidine hydrogen bond network of the R128 residue forms a dynamic stress transfer chain. The R128H mutation reduces the side chain movement by 40%, increases the electrostatic interaction gradient by 2.3 times, and increases the catalytic efficiency to 3.8 times that of the wild type [63]. In the R128K/M113W double mutant, the compressive stress of the tryptophan indole ring and the tensile stress of the lysine ε-amino group act synergistically to increase the polarizability of the cyano group and increase the catalytic rate constant by 5.2 times [63]. These findings demonstrate the remarkable advantage of rationally engineered catalytic systems in achieving high enantioselectivity (ee > 98%) and significantly improved catalytic efficiency. However, despite these improvements, a key limitation remains. Molecular dynamics trajectory analysis found that there is a hydrogen bond network reconstruction phenomenon in the product release stage, the lifetime of the key hydrogen bond is reduced, and the non-target enantiomer is easily racemized and degraded during the reaction, resulting in low productivity [64,65]. This drawback—product racemization during release—poses a significant challenge, as it compromises optical purity over time and reduces the overall yield of the reaction.

#### 4.2.2. Equilibrium Control in Lipase-Mediated Dynamic Resolution

The stereoselective hydrolysis of methyl/ethyl mandelate esters by lipases (EC 3.1.1.3) and esterases (EC 3.1.1.1) enables the conversion of racemic ester substrates into single-configuration MA through a dynamic kinetic resolution system. A representative process employs immobilized *Candida antarctica* lipase B in an organic–aqueous biphasic system for continuous catalysis. Precise regulation of pH and temperature enhances product enantiomeric excess (ee) to >98%, with reusable enzyme catalysts [66,67]. This system not only achieves high optical purity (ee > 98%) but also benefits from the use of immobilized enzymes, which are reusable and cost-effective for industrial applications. Mechanistic optimization involves tuning the elastic modulus of the immobilized matrix’s porous structure to balance conformational of the enzyme and substrate mass transfer kinetics. However, a major challenge arises from the delicate balance between esterification and hydrolysis, as the system requires stringent control over the dynamic equilibrium. This leads to an inherent trade-off between achieving high substrate conversion and maintaining enantiomeric purity. This technical bottleneck limits overall process efficiency and remains a key hurdle in large-scale application.

#### 4.2.3. Synergistic Mechanism of Dehydrogenase-Laccase Cascade Systems

The synergistic catalysis by dehydrogenases and laccases overcomes the limitations and drawbacks associated with redox-involved multi-cascade reactions, breaking through the constraints of traditional pathways [68]. During the reduction in phenylglyoxylic acid, the mechanical allosteric effects within the coenzyme-binding domain significantly influence hydride ion transfer efficiency, leading to a theoretical yield of 100% and offering a promising solution for electron transfer bottlenecks in complex biocatalytic systems. The coenzyme regeneration system is realized through laccase (EC 1.10.3.2)-mediated redox cycling [59,68]. As a tetranuclear copper oxidase, laccase modulates the activation energy barrier for oxygen reduction via geometric strain in its copper ion clusters. This mechanism simultaneously accomplishes oxygen reduction and ferrocyanide oxidation, effectively resolving electron transfer imbalances in multi-enzyme cascade systems [69,70]. However, despite these advantages, practical implementation faces significant challenges. The coordination of multiple enzymes requires precise control over reaction timing and stoichiometry, while modulating geometric strain in laccase’s copper clusters for optimal activity remains technically demanding.

#### 4.2.4. Microenvironment Reconfiguration in Microbial Cell Factories

In engineered strain construction, modulating the mechanical response threshold of the cell membrane tension-sensing system optimizes substrate transmembrane transport efficiency. Real-time monitoring of mechanical stress fluctuations at key nodes in metabolic pathways via fluorescence resonance energy transfer technology enables dynamic equilibrium of metabolic flux distribution [71]. Integration of exogenous enzymes into microbial cells, coupled with metabolic pathway optimization, enables one-pot biosynthesis of MA with high product titer and optical purity, demonstrating significant advantages in streamlined production processes [72,73]. In the reconstruction of the microenvironment of microbial cell factories, cell membrane mechanical sensing has become a key strategy for optimizing transmembrane transport of substances by regulating the gating threshold of mechanically sensitive channels. Studies have shown that gene editing of membrane tension-sensitive proteins can accurately regulate the dynamic response of cells to external fluid shear forces. Lv et al. found that when the MscL channel was knocked out, the cell membrane needed to withstand a greater tension (from 8 to 12 mN/m) before the “safety valve” would open [74]. This modification not only reduced the leakage of the energy molecule ATP under non-ideal conditions, but also triggered the opening of the residual channel through osmotic pressure changes when needed, increasing the transmembrane efficiency of the target substrate by 3.2 times [74]. Nie et al. rationally designed the MscCG2 channel of *Corynebacterium glutamicum* and screened out the F68W/V77A double mutant. The sensitivity of the MscCG2 channel to shear force increased by nearly one-fold. The mutation made the channel structure more susceptible to conformational changes, thereby increasing the efficiency of glutamate production by 3.7 times [75]. Combined with the membrane lipid phase transition temperature control strategy proposed by Balleza’s team, after increasing the proportion of palmitic acid in the cell membrane to 35%, the rigidity of the membrane structure was significantly enhanced, and the substrate-directed uptake efficiency was increased to 89% [76]. Kawasaki et al. further confirmed that overexpression of modified MscCG2 can maintain steady-state tension in the cell membrane during fermentation, avoiding membrane rupture and increasing L-glutamate production by 58% [77]. However, these advancements rely heavily on advanced genetic and metabolic engineering techniques, which pose significant technical challenges and may limit their broad applicability in industrial settings. This mechanical-metabolic coupling mechanism provides a molecular biomechanical basis for the intelligent adaptation of industrial strains.

**Table 5:** Comparative Analysis of MA Biosynthesis Methods.

Category	Method/ Mechanism	Key Process	Advantages	Limitations	References
Biosynthesis	Nitrilase Catalysis	Active-site engineering to enhance stress fields	High enantioselectivity (ee > 98%), improved catalytic efficiency	Product racemization during release, low productivity	[56,61,65]
	Lipase-mediated Resolution	Dynamic kinetic resolution with immobilized lipases	Reusable enzymes, high ee (>98%)	Trade-off between substrate conversion and optical purity	[66,67]
	Dehydrogenase-Laccase Cascade	Reductive amination and laccase-mediated cofactor regeneration	Theoretical 100% yield, resolves electron transfer bottlenecks	Complex multienzyme coordination, copper cluster strain modulation challenges	[59,68,69,70]
	Microbial Cell Factories	Membrane tension engineering and metabolic flux control	High titer/optical purity, one-pot biosynthesis	Requires advanced genetic/metabolic engineering	[71,72,73,74,77]

## 5. Advances in the Biosynthesis of MA

### 5.1. Synthesis of (S)-MA and (R)-MA from Mandelonitrile

**Figure 3:**
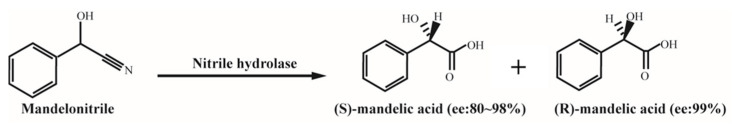
Synthesis of (S)-MA and (R)-MA from Mandelonitrile.

In chiral drug synthesis, the divergent stereoselectivity of nitrilase catalytic systems reflects deep-rooted biomechanical mechanisms. Studies have shown that most nitrilases achieve stereoselective substrate binding through the specific three-dimensional architecture of their active sites. This biomechanical feature, based on the intermolecular force network within enzyme–substrate complexes, leads to the preferential production of (R)-MA [64]. However, naturally occurring (S)-selective nitrilases exhibit significant technical limitations due to mechanical instability within their active centers. Steric hindrance during substrate binding inhibits effective enantiomeric discrimination, resulting in suboptimal ee values. The general biosynthetic pathways for both (S)- and (R)-MA derived from mandelonitrile are schematically illustrated in Figure 3, providing a conceptual framework for understanding enzymatic stereoselectivity.

From a biomechanical standpoint, the condensation of benzaldehyde with hydrogen cyanide to form mandelonitrile can be seen as a molecular orbital energy-level alignment process. While traditional chemical catalysts function by constructing low-strain transition-state pathways [55,56], modern enzymatic strategies harness the elastic deformation energy stored in protein conformations, using mechanical forces generated within enzymes to drive reactions. For example, when using *Alcaligenes faecalis* ATCC 8750 whole-cell catalysts [55], the osmotic pressure gradient across the cell wall creates a mechanical microenvironment that substantially enhances substrate transport, achieving a 99% conversion to (R)-MA. This insight offers theoretical guidance for optimizing hydrodynamic conditions in industrial-scale processes.

To overcome the mechanical instability underlying the (S)-selectivity limitations, molecular dynamics simulations have identified key amino acid residues (M113, R128) that regulate the mechanics of the active site via side-chain conformations. Site-directed mutagenesis has been applied to enhance hydrogen-bond networks, thereby optimizing strain distribution in the transition state and increasing the (S)-MA ee value from 80% to 91% [62]. This protein-mechanical engineering strategy successfully directs intramolecular stress fields toward improved stereoselectivity.

In the biosynthesis of glycolic acid enantiomers, the mechanical synergy of a multienzyme cascade system significantly enhances the precision of stereoselective control. A dual-enzyme system composed of cassava (S)-hydroxynitrile lyase and *Pseudomonas fluorescens* nitrilase forms an efficient substrate-transfer channel through intermolecular forces, functioning like a molecular-level “conveyor belt” and achieving an ee value of 98.0% [78]. It is worth noting that by-product formation originates from “attack deviation” at the nitrilase active site, much like error accumulation in mechanical processing [79]. Introducing an amidase to create a three-enzyme system enables directional energy flow through mechanical signal transduction: the mechanical energy released by nitrilase during catalysis triggers conformational changes in amidase—like water driving a waterwheel—and reintroduces otherwise dissipated energy into the reaction pathway, significantly enhancing by-product conversion efficiency [80,81].

The interfacial forces and energy transfer efficiency between cassava (S)-hydroxynitrile lyase (D-HNL) and nitrile hydrolase (NLase) directly affect the conversion kinetics from cyanohydrin to carboxylic acid. Zhou et al. revealed through molecular dynamics simulations that electrostatic complementarity between the flexible loop region of the D-HNL active site and the substrate-binding domain of NLase can form a dynamic hydrogen-bond network, which optimizes the molecular orientation of the cyanohydrin intermediate, reduces binding free energy by 3.8 kcal/mol, and significantly shortens the substrate transfer distance [82]. Based on this, the researchers applied rational design to reinforce the salt bridge between Arg137 of D-HNL and Glu294 of NLase, thereby reducing the dissociation constant of the enzyme complex to 0.32 μM, which is 6.5 times lower than that of the wild type [82].

The construction of an inter-enzyme force transmission channel requires a balance between structural rigidity and dynamic coupling. Dadashipour et al. found that enhancing the rigidity of the β-barrel structure through a Gly205Pro mutation—like armoring the enzyme molecule—increased the hydrophobic coupling efficiency with nitrilase by 40% and raised the intermediate transfer rate to 1.8 s^−1^, effectively mitigating energy loss caused by asynchronous enzyme conformational changes in conventional systems [83]. In addition, Sheldon’s team developed a “molecular anchoring” strategy, using the genetically encoded SpyTag/SpyCatcher system to precisely fix the dual enzymes onto a magnetic carrier, much like a magnet attracting metal, stabilizing the inter-enzyme distance at around 4.7 nanometers. This design created a rigid mechanical stress conduction channel, boosting catalytic efficiency by 2.3-fold, akin to building a high-speed track for enzymatic reactions [84].

### 5.2. Synthesis of (S)-MA and (R)-MA from MA Esters

**Figure 4:**
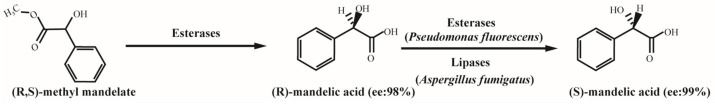
Synthesis of (S)-MA and (R)-MA from (R,S)-methyl mandelate.

The stereoselective catalysis by lipases and esterases fundamentally represents a biomechanically driven molecular recognition process. In the kinetic resolution of (S)-MA esters, the enzyme active center generates a mechanically asymmetric field through vectorial superposition of hydrogen-bond networks and van der Waals forces, precisely regulating the selective formation of the (R)-enantiomer. Esterases derived from *Pseudomonas* sp. leverage geometric constraint effects in their substrate-binding pockets during the hydrolysis of (R,S)-methyl mandelate. Substrate molecules experience torque forces exerted by active-site residues, inducing specific cleavage of the (R)-configured substrate to achieve (R)-MA synthesis with ee > 98% [57,58]. Research by the Chen group further revealed that the ammonolysis reaction catalyzed by *Candida antarctica* lipase exhibits unique dynamic mechanical properties [85]. Its α-helical structure generates periodic mechanical forces through conformational changes to drive ammonolysis, achieving 99% ee for (R)-MA. Figure 4 provides a schematic overview of the enzymatic resolution pathways leading to both (S)- and (R)-MA from (R,S)-methyl mandelate. This mechano-chemical coupling mechanism introduces novel design dimensions for enzymatic resolution.

In (S)-MA synthesis, the mechanical response characteristics of enzymes demonstrate finer regulatory precision. Esterases from *Pseudomonas fluorescens* and recombinant lipases from *Aspergillus fumigatus* achieve stereospecific hydrolysis through stress field reconfiguration in their active pockets [57]. Molecular dynamics analysis indicates that side-chain displacements of esterase residues during substrate binding induce localized shear stress, forcing the (R)-enantiomer into mechanically unstable conformations, thereby selectively retaining (S)-MA (ee > 99%). This method exhibits significant industrial potential and validates the technical superiority of ester hydrolysis/esterification systems in producing optically pure MA.

### 5.3. Synthesis of (S)-MA and (R)-MA from (S)-Phenyl-1,2-glycol

**Figure 5:**
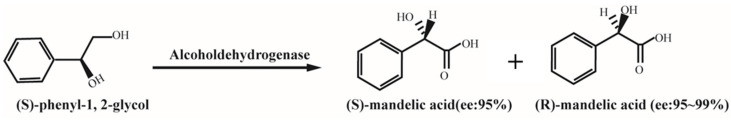
Synthesis of (S)-MA and (R)-MA from (S)-Phenyl-1,2-glycol.

In the chiral biosynthesis of MA from (S)-phenyl-1,2-glycol, the stereoselectivity of enzymes fundamentally arises from a biomechanically driven molecular recognition process. The catalytic system centered on aldehyde-ketone dehydrogenases generates a mechanically asymmetric field at the active site through synergistic hydrogen-bond networks and van der Waals interactions, enabling directional selection of the (R)-configured product. Although *Bordetella parapertussis* catalyzes the degradation of phenyl-1,2-glycol to produce (R)-MA with an ee value of 95% [23], the optical purity remains limited. Molecular dynamics simulations reveal that substrate molecules undergo asymmetric stress field effects during binding, a mechanical response mechanism that constitutes the physical basis of stereoselectivity. A schematic depiction of this biosynthetic pathway is presented in Figure 5, which illustrates the transformation routes from (S)-phenyl-1,2-glycol to both (R)- and (S)-MA.

Biomechanical engineering plays a pivotal role in process intensification. The *Brevibacterium lutescens* CCZU12-1 strain developed by He et al. [86], optimized via directed evolution, enhances intramolecular mechanical transmission pathways, achieving a breakthrough (R)-MA ee value of 99.9%. In immobilized processes, the porous structure of calcium alginate carriers regulates elastic modulus to form protective mechanical barriers, effectively buffering shear stress impacts in the reaction system. This elevates the product-to-biocatalyst ratio to 27.94 g/g dcw. Dual optimization through process innovation—employing a fed-batch strategy—enables (R)-MA accumulation up to 172.9 mM. This synthesis protocol not only ensures efficient biocatalyst recycling but also demonstrates significant industrial applicability.

For (S)-MA synthesis, the aldehyde-ketone dehydrogenase from *Pseudomonas fluo-rescens* N3 exhibits unique mechanical regulation [87]. Its catalytic center’s α-helical structure generates periodic mechanical forces through conformational changes to drive (S)-enantiomer formation (ee 95%). Compared to conventional hydrolysis pathways, this redox route innovatively exploits secondary alcohol dehydrogenase characteristics, reducing transition-state activation energy barriers to circumvent byproduct formation, thereby establishing a foundation for green synthesis of chiral MA.

### 5.4. Synthesis of (S)-MA and (R)-MA from Styrene, Bio-Based L-Phenylalanine, and Renewable Feedstocks

In the biosynthesis of (S)-MA, the modular design of cascade catalytic systems is driving paradigm shifts in chiral molecule manufacturing. Sun pioneered the reconstruction of *Escherichia coli*’s L-phenylalanine metabolic network by introducing 4-hydroxymandelate synthase (*Ao*HmaS) from *Amycolatopsis orientalis*, establishing a glucose-to-(S)-MA pathway (initial titer: 0.092 g/L) [88]. Notably, while *Ao*HmaS naturally acts on 4-hydroxyphenylpyruvate, its active site exhibits conformational adaptability to non-specifically convert phenylpyruvate [89]. Knockout of competing metabolic genes enhanced flux, elevating (S)-MA production to 0.74 g/L within 24 h, though the low catalytic efficiency of HmaS remains a critical bottleneck.

To address this limitation, researchers explored HmaS variants from diverse sources. Guided by molecular dynamics simulations, a quadruple mutant (A199V/Q206R/I217V/K337Q) of 4-hydroxymandelate synthase (*At*HmaS) from *Actinoplanes teichomyceticus* demonstrated 2.4-fold higher activity than the wild type [90]. Structural analysis revealed that the hydrophobic pocket formed by Ile217 stabilizes the substrate’s phenyl ring, mirroring the functional homology of Ile216 in *Ao*HmaS [91], providing a molecular blueprint for further enzyme engineering.

Reifenrath achieved breakthroughs in eukaryotic systems. By mutating the substrate-binding domain (S201V) of *Ao*HmaS in *Saccharomyces cerevisiae* to block 4-hydroxyphenylpyruvate binding, they redirected catalysis toward phenylpyruvate. Leveraging yeast’s metabolic regulation, (S)-MA production reached 0.236 g/L from glucose—a 200-fold improvement over initial systems [72]. This work not only highlights the potential of eukaryotic platforms in chiral synthesis but also establishes a foundation for modular construction of complex MA derivatives [92].

**Figure 6:**
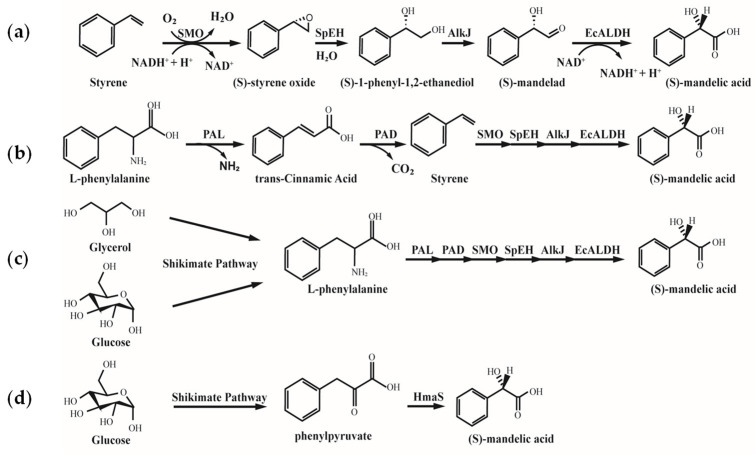
Synthesis of (S)-MA from Styrene, L-Phenylalanine, and Renewable Feedstocks [89,93]. (**a**) Biosynthesis from Styrene; (**b**) Biosynthesis from L-Phenylalanine; (**c**) Biosynthesis from Glycerol and Glucose; (**d**) Biosynthesis from Glucose; SMO: styrene monooxygenase; SpEH: epoxide hydrolase; AlkJ: alcohol dehydrogenase; EcALDH: acetaldehyde dehydrogenase; PAL: phenylalamine ammonia lyase; PAD: phenylacrylic acid decarboxylase; HmaS: hydroxymandelate synthase.

For industrial applications, Lukito developed a four-enzyme cascade system in engineered *E. coli* LZ37, converting styrene to (S)-MA at 18–21.9 g/L (118–144 mM) with 72–79% molar conversion (Figure 6) [93]. The process maintained stability at 1.5 L scale, yielding 21.3 g/L (70% recovery). Biomechanically, interfacial tension optimization in biphasic solvents reduced mass transfer resistance, enabling cumulative production of 49.1 g/L (328 mM, 82% yield) over four batches, advancing continuous industrial production.

Expanding substrate versatility, a six-step cascade system in *E. coli* LZ37 modularized amino acid metabolism with chiral synthesis, directly converting L-phenylalanine to (S)-MA (24.3 g/L, 160 mM, 80% yield). Remarkably, integrating renewable carbon modules achieved 10 g/L (63 mM) from glycerol and 8 g/L (52 mM) from glucose, enabling biomass-to-chemical carbon flux redirection (Figure 6) [93]. This strategy overcomes fossil feedstock dependence and exemplifies green biosynthesis through metabolic network reconfiguration.

In (R)-MA synthesis, traditional substrate toxicity arises from osmotic gradient-induced mechanical stress. Sun rebalanced cellular mechanics via synthetic biology: heterologous expression of *A. orientalis* HmaS stabilized substrate binding through hydrogen network redesign, yielding 0.092 g/L (S)-MA [88]. Introducing *Streptomyces coelicolor* hydroxymandelate oxidase (HmO) and *Rhodotorula graminis* D-mandelate dehydrogenase (DMD) created an (R)-MA biosystem (0.68 g/L). Deleting competing pathways redirected carbon flux by reshaping intracellular stress fields.

**Figure 7:**
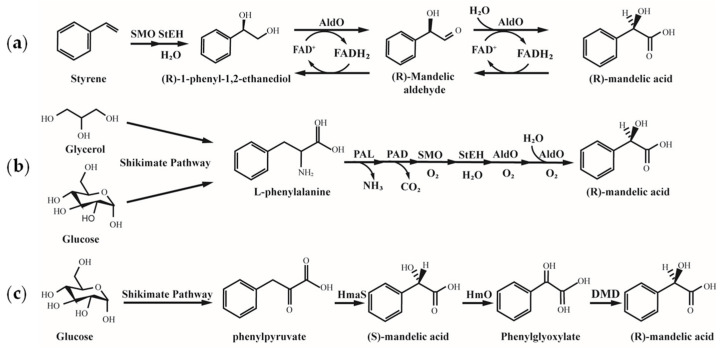
Synthesis of (R)-MA from Styrene and Renewable Feedstocks [73,88]. (**a**) Biosynthesis from Styrene; (**b**) Biosynthesis from Glycerol and Glucose; (**c**) Biosynthesis from Glucose; SMO: styrene monooxygenase; StEH: epoxide hydrolase; AldO: alditol oxidase; PAL: phenylalamine ammonia lyase; PAD: phenylacrylic acid decarboxylase; HmaS: hydroxymandelate synthase; HmO: hydroxymandelate oxidase; DMD: D-mandelate dehydrogenase.

Lukito innovated a three-enzyme “epoxidation–hydrolysis–dioxygenation” cascade to synthesize (R)-MA directly from styrene (1.52 g/L, >99% ee) (Figure 7) [73]. A five-enzyme system integrating deamination and decarboxylation modules achieved whole-cell conversion of L-phenylalanine to (R)-MA, with engineered *E. coli* producing 0.913 g/L (>99% ee) from glucose—a record for natural resource-based routes (Figure 7). This technology enables sustainable chiral drug production via renewable feedstocks and green catalysis.

### 5.5. Synthesis of (R)-MA from (S)-MA

In the biosynthesis of (R)-MA, the biomechanical properties of enzyme molecules and the engineered mechanical design of catalytic systems are pivotal in overcoming challenges associated with chiral control. *Burkholderia cepacia* lipase demonstrates a unique molecular recognition mechanism, whereby its active site maintains >99% stereoselectivity even under low concentrations of (S)-MA, through precise hydrogen-bond networks and steric hindrance effects within intermolecular force frameworks [94]. In contrast, *Pseudomonas stutzeri* lipase achieves high tolerance and chiral discrimination by resisting conformational changes induced by fluid shear stress, a property attributed to its tertiary structure under high substrate concentrations [95]. These findings provide valuable biomechanical insights for hydrodynamic optimization in industrial-scale bioreactors.

**Figure 8:**
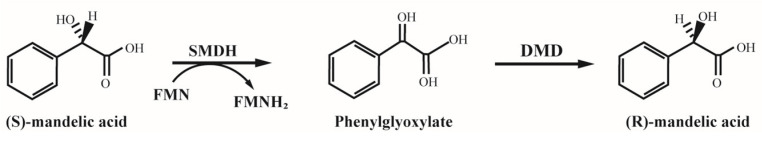
Synthesis of (R)-MA from (S)-MA. SMDH: S-mandelate dehydrogenase; DMD: D-mandelate dehydrogenase.

From a biomechanical energy conversion perspective, the catalytic process of NADH-dependent (R)-MA dehydrogenase exhibits sophisticated mechanical synergy. The enzyme uses a flexible hinge region in its active site to facilitate directional induced-fit binding of phenylglyoxylic acid, with molecular docking to the cofactor NAD^+^ involving dynamic force modulation (Figure 8) [96]. Mechanical modification of the enzyme via protein engineering significantly enhances system stability, thereby laying a biomechanical foundation for continuous bioreactor operation.

In the S-mandelate dehydrogenase (SMDH) system, a key challenge lies in energy dissipation during cofactor regeneration, particularly due to interrupted redox potential transmission. Researchers found that the copper ion cluster at the laccase active site behaves like a precise molecular spring, where geometric deformation directly regulates electron transfer efficiency. The T1 copper center establishes a bioelectrochemical gradient mediated by ferrocyanide, enabling a pathway for cofactor FMNH_2_ regeneration. Durao et al. discovered that axial ligand shifting at His419 in *Bacillus subtilis* CotA laccase causes the T1 copper coordination geometry to deform—from tetrahedral to triangular pyramidal—which optimizes the electron pathway like adjusting an antenna. This reduces the Cu–S (Met502) bond length by 12%, lowers the electron tunneling energy threshold by 1.8 eV, and decreases energy consumption during oxygen reduction [97]. Hitaishi et al. further revealed via single-molecule force spectroscopy that when the laccase is tilted 16° on the electrode surface, the T2/T3 trinuclear copper cluster behaves like a compressed spring, inducing a 5.3% compressive strain and enhancing the electrostatic attraction at the oxygen-binding pocket. This lowers the activation barrier for oxygen reduction and significantly increases current output [98]. Moreover, introducing the L386A mutation to enhance copper cluster flexibility acts like lubricating the molecular spring, improving energy transfer efficiency by 38% and increasing catalytic turnover frequency beyond 400 s^−1^ [99].

The dynamic coupling of cofactor regeneration relies on the mechanical responsiveness of the copper ion cluster. Janusz et al. pointed out that changes in the oxidation state of T1 copper during the laccase catalytic cycle trigger the T2/T3 cluster to undergo periodic expansion–contraction. This nanovibration is transmitted through the protein scaffold, functioning like a molecular pump, and increases proton transfer efficiency by 67% [100]. In the chiral resolution of (S)-substrates, SMDH from *Pseudomonas putida* ATCC 12633 selectively oxidizes (S)-MA to phenylglyoxylic acid, reducing cofactor FMN to FMNH_2_ and thereby enabling directional enrichment of (R)-MA [101]. However, FMNH_2_ regeneration remains a bottleneck due to redox potential discontinuity [102]. Wang’s team addressed this using a dehydrogenase–laccase dual system. Laccase, a copper-containing phenol oxidase, oxidizes ferrocyanide, generating water and supporting in situ FMN regeneration. Cycling between ferrocyanide/ferricyanide establishes a stable energy transfer pathway [59,60], allowing continuous stereoselective transformation with optical purity exceeding 99% ee.

Notably, current dual-enzyme systems face engineering challenges: free enzymes show poor stability under flow conditions, while dialysis-based immobilization leads to catalyst recovery issues. As shown in the latest advances summarized in Table 6, future approaches may leverage the structural mechanics of biofilms, designing immobilized enzyme composites with carriers that maintain conformational flexibility while improving shear resistance. These biomechanical strategies could help overcome industrial application barriers.

## 6. Prospect

### 6.1. From Enzyme Engineering to System Integration: Technological Evolution

Although traditional chemical synthesis methods can produce both (R)-MA and (S)-MA, they face critical limitations, including the use of highly toxic cyanides, complex procedures, poor stereoselectivity, and excessive byproduct formation. These issues not only increase environmental costs but also hinder scalable production and the transition toward greener manufacturing processes [103]. In this context, biosynthetic approaches have emerged as a key solution for overcoming traditional synthesis bottlenecks, offering advantages in efficiency, environmental sustainability, and superior stereoselectivity.

Recent innovations in biocatalytic technology focus on the design and optimization of enzymatic cascade reaction systems [104]. Researchers employ synthetic biology tools to construct artificial enzyme cascades in engineered cells, integrating natural metabolic pathways with rationally designed reaction modules. This enables efficient, directional conversion of renewable substrates into MA. By harnessing the mechanical transduction properties of native pathways (e.g., ATP hydrolysis-driven conformational changes) and the coupling mechanisms of engineered modules (e.g., substrate channeling specificity), high-efficiency substrate-to-product conversion is achieved [105]. Genomic integration technologies and promoter engineering allow precise regulation of multi-enzyme expression levels and spatiotemporal distribution within cascades, significantly enhancing overall catalytic efficiency [93,106].

Advances in enzyme engineering further underpin biosynthesis breakthroughs. Directed evolution screens for hyperactive, stable mutant enzymes, while rational design and computational prediction tools optimize substrate-binding affinity and stereoselectivity. Modularization strategies reduce inhibitory effects from intermediate accumulation by decoupling and reconfiguring enzymatic reaction steps [107]. To address metabolite toxicity, biphasic catalytic systems and in situ product removal technologies mitigate cellular inhibition by intermediates or products, prolonging catalytic system longevity [108]. These dynamic regulation systems, grounded in biomechanical principles, offer novel technical frameworks for sustaining complex biocatalytic processes.

### 6.2. From Laboratory to Industrialization: Potential Challenges

Despite the huge potential of biosynthesis technology, its industrial-scale implementation still faces multiple challenges. In terms of coordinating multi-enzyme systems, the kinetic compatibility among different enzymes can vary significantly, and the optimal conditions for each enzyme must be balanced in complex cascade reactions. This places greater demands on understanding enzyme dynamic equilibrium mechanisms. Changes in the conformational rigidity of enzyme active sites may lead to compatibility conflicts under optimal reaction conditions (such as pH, temperature, and ionic strength), thereby requiring more in-depth studies of dynamic equilibrium behavior [109,110].

The issue of transmembrane transport efficiency is also prominent. The biomechanical properties of cell membranes limit the transport rate of intermediates, causing imbalances in metabolic flux. Therefore, the transport mechanisms must be optimized to improve overall productivity [108]. In addition, some synthetic pathways still depend on chemical intermediates, which contradicts the goals of green synthesis. Thus, the development of fully biosynthetic routes is necessary. The accumulation of certain precursors in cells may lead to feedback inhibition, and the high cost of exogenous cofactor supplementation undermines economic feasibility.

At the level of process optimization and regulation, it is crucial to integrate continuous production technologies with dynamic metabolic regulation strategies to enable precise control of reaction conditions and improve resource utilization efficiency. Catalyst design must move beyond traditional static optimization, and instead focus on intelligent response systems. These systems can leverage artificial intelligence to predict structure–function relationships, enabling the development of catalysts that dynamically adapt to changing environments [111]. In the field of intelligent biomechanical systems, there is a need to explore force-responsive enzymes, such as light- or heat-controlled conformational switch enzymes, to precisely regulate catalytic.

Cross-scale integration strategies require the analysis of global mechanical regulatory networks, combining transcriptomic and mechanical phenotype data to optimize biosynthetic processes. Additionally, constructing biochemical hybrid systems involves the co-design of interface buffer layers and molecular recognition sites to achieve mechanical compatibility between biocatalysis and chemical synthesis, while balancing reaction activity and conformational stability.

### 6.3. The Future Directions of Green Biosynthesis

To achieve industrial breakthroughs in the biosynthesis of MA, synergistic innovations across three key dimensions—raw materials, catalysts, and processes—are essential. At the raw material level, emphasis should be placed on the mechanical compatibility of biomass feedstocks. Developing biosynthetic pathways derived from renewable resources remains a core strategy for minimizing the environmental footprint of green synthesis. In catalyst design, it is critical to move beyond traditional static optimization in enzyme engineering and focus on constructing mechanically dynamic, responsive systems. Leveraging artificial intelligence to predict enzyme structure–function relationships will enable the design of intelligent catalysts capable of dynamically adapting to environmental changes [112]. Process optimization necessitates the integration of continuous production engineering with dynamic metabolic regulation strategies to achieve precise control over reaction conditions and enhance resource utilization efficiency.

In the future development of green biosynthesis, intelligent biomechanical systems and cross-scale integration strategies will become key innovations [111]. Regarding intelligent biomechanical systems, it is particularly important to develop force-responsive intelligent enzymes (such as light- or heat-controlled conformational switch enzymes). These enzymes can sense external mechanical stimuli and dynamically regulate catalytic activity, thereby achieving precise control of reaction conditions and improving catalytic efficiency and product selectivity. For example, by regulating enzyme conformational changes through light-controlled switches, specific metabolic pathways can be activated or inhibited, providing new tools for dynamic metabolic regulation. The cross-scale integration strategy emphasizes the analysis of the global mechanical regulatory network of synthetic pathways from the perspective of single-cell mechanomics. Combined with transcriptome–mechanical phenotype association analysis, the deep correlation between cellular transcriptional states and mechanical phenotypes can be revealed, providing multi-scale data support for optimizing biosynthetic processes [113,114]. For instance, by analyzing transcriptomic changes under different mechanical conditions, key regulatory genes can be identified, and the mechanical properties of cells can be modulated through genetic engineering to improve the overall efficiency of the synthetic pathway [114].

It is particularly noteworthy that the innovative construction of biochemical hybrid systems, through the synergistic design of interfacial buffer layers and molecular recognition sites, has achieved mechanical compatibility between biocatalysis and chemical synthesis. This cross-domain fusion strategy not only preserves the high reactivity of transition metal catalysts but also maintains the three-dimensional conformational stability of enzymes [115]. By simplifying synthetic pathways and eliminating the need for highly toxic reagents such as cyanides, this approach demonstrates unique advantages in bridging the gap toward industrial-scale green chemical manufacturing.

## 7. Conclusions

The biosynthesis of MA is decisively transitioning from laboratory research to industrial implementation. Despite persistent challenges including kinetic incompatibilities in enzymatic systems, suboptimal metabolic regulation precision, and scale-up cost barriers, its transformative potential in overcoming chiral synthesis bottlenecks and revolutionizing green manufacturing is unequivocal. Critically, the integration of biomechanics with synthetic biology is reshaping this field: biomechanical analysis of microbial responses in bioreactors enables targeted environmental optimization for enhanced fermentation efficiency, while molecular dynamics simulations reveal enzyme–substrate complex stability patterns, providing a rational structural framework for engineering high-activity biocatalysts—this rational enzyme engineering via molecular dynamics simulations lays the computational groundwork for constructing responsive systems (though such systems remain nascent in molecular dynamics biosynthesis). These advances collectively underpin intelligent, responsive biosynthesis systems. This evolution toward mechanically informed bio-manufacturing will not only redefine MA production paradigms but also accelerate the sustainable transition of chiral chemical synthesis. By delivering efficient, eco-friendly solutions to pharmaceutical and fine chemical industries, it establishes a tripartite synergy of economic viability, environmental stewardship, and social responsibility.

## Figures and Tables

**Table 4:** Comparative Analysis of MA Chemical Synthesis Methods.

Category	Method/ Mechanism	Key Process	Advantages	Limitations	References
Chemical Synthesis	Traditional Methods	Cyanohydrin Hydrolysis; α-Haloacetophenone Hydrolysis	Mature technology, widely applicable	High toxicity (cyanides/halogens), high energy input, hazardous wastewater	[49,50,51]
	Phase-transfer Catalysis	Biphasic reaction with quaternary ammonium salts under ambient conditions	Mild conditions, avoids strong oxidants/reductants	Catalyst leaching, poor recyclability, long reaction time	[52]
	Asymmetric Synthesis	Chiral ligands for single-enantiomer production	High optical purity (ee > 98%)	High cost of chiral catalysts, inert atmosphere required	[53]
	Optical Resolution	Racemate synthesis and chiral separation	Obtains enantiopure products	Low efficiency, resolving agent recovery challenges, low yield	[54]

**Table 6:** Advances in Biosynthesis of MA.

Category	Brief Description	Examples	References
Synthesis from Mandelonitrile	*Stereoselective nitrilase* systems optimized via enzyme engineering and molecular dynamics	M113F/R128K mutants (S)-MA; Dual-enzyme system (cassava D-HNL)	[55,63,77]
Synthesis from MA Esters	Lipase/esterase-mediated dynamic resolution with mechanical asymmetry in active sites	*Pseudomonas* sp. esterase (R)-MA; *Candida antarctica* lipase ammonolysis	[57,58,85]
Synthesis from (S)-Phenyl-1,2-glycol	Aldehyde-ketone dehydrogenase systems with mechanical stress modulation	*Brevibacterium lutescens* CCZU12-1 (R)-MA; dehydrogenase (S)-MA	[23,86,87]
Synthesis from Styrene/Renewables	Modular cascade systems for green synthesis using engineered microbes	*E. coli* LZ37; *Ao*HmaS mutant; Six-step *E. coli* system	[71,72,88,89,90]
(R)-MA from (S)-MA	Enzyme cofactor regeneration and redox potential optimization	*Burkholderia cepacia* lipase; Dehydrogenase-laccase dual system; Laccase T1 copper mutants	[59,60,94]

## Data Availability

No new data were created or analyzed in this study.

## References

[1] Choińska R., Dąbrowska K., Świsłocka R., Lewandowski W., Świergiel A.H. (2021). Antimicrobial Properties of Mandelic Acid, Gallic Acid and their Derivatives. Mini Rev. Med. Chem..

[2] Martinkova L., Kren V. (2018). Biocatalytic production of mandelic acid and analogues: A review and comparison with chemical processes. Appl. Microbiol. Biotechnol..

[3] Dehghani Z., Akhond M., Jangi S.R.H., Absalan G. (2024). Highly sensitive enantioselective spectrofluorimetric determination of R-/S-mandelic acid using l-tryptophan-modified amino-functional silica-coated N-doped carbon dots as novel high-throughput chiral nanoprobes. Talanta.

[4] Norvaiša K., O’bRien J.E., Osadchuk I., Twamley B., Borovkov V., Senge M.O. (2022). Importance of molecular symmetry for enantiomeric excess recognition by NMR. Chem. Commun..

[5] Han Y., Kou M., Zhang H., Qiu H., Shi Y.P. (2025). Chiral fluorescent carbon dots for tyrosine enantiomers: Discrimination, mechanism and cell imaging. Sens. Actuators B Chem..

[6] Sakai R., Mato Y., Ishimaru H., Ogata K., Kurose S., Ozawa S., Umeda S., Tsuda K., Satoh T., Kakuchi T. (2024). Colorimetric Sensing of Chirality Based on Synergistic Effect of Multiple Chiral Amide Receptors Consecutively Organized along Poly (phenylacetylene) Backbone. Macromolecules.

[7] Świsłocka R., Świderski G., Nasiłowska J., Sokołowska B., Wojtczak A., Lewandowski W. (2023). Research on the Electron Structure and Antimicrobial Properties of Mandelic Acid and Its Alkali Metal Salts. Int. J. Mol. Sci..

[8] Matejczyk M., Ofman P., Świsłocka R., Parcheta M., Lewandowski W. (2022). The study of biological activity of mandelic acid and its alkali metal salts in wastewaters. Environ. Res..

[9] Zhang Y., Wang X., Shi H., Siddique F., Xian J., Song A., Wang B., Wu Z., Cui Z.N. (2024). Design and Synthesis of Mandelic Acid Derivatives for Suppression of Virulence via T3SS against Citrus Canker. J. Agric. Food Chem..

[10] Kolano M., Ohnmacht B., Lemmer A., Kraume M. (2024). How Fluid Pseudoplasticity and Elasticity Affect Propeller Flows in Biogas Fermenters. Chem. Ing. Tech..

[11] Wang Z.K., Gong J.S., Feng D.T., Su C., Li H., Rao Z.M., Lu Z.M., Shi J.S., Xu Z.H. (2023). Geometric Remodeling of Nitrilase Active Pocket Based on ALF-Scanning Strategy To Enhance Aromatic Nitrile Substrate Preference and Catalytic Efficiency. Appl. Environ. Microbiol..

[12] Zeng X.H., Du H., Zhao H.M., Xiang L., Feng N.X., Li H., Li Y.W., Cai Q.Y., Mo C.H., Wong M.H. (2020). Insights into the binding interaction of substrate with catechol 2, 3-dioxygenase from biophysics point of view. J. Hazard. Mater..

[13] Kell D.B. (2018). Control of Metabolite Efflux in Microbial Cell Factories: Current Advances and Future Prospects in Fermentation Microbiology and Biotechnology.

[14] Christianson J.C., Jarosch E., Sommer T. (2023). Mechanisms of substrate processing during ER-associated protein degradation. Nat. Rev. Mol. Cell Biol..

[15] Ghoula M., Janel N., Camproux A., Moroy G. (2022). Exploring the Structural Rearrangements of the Human Insulin-Degrading Enzyme through Molecular Dynamics Simulations. Int. J. Mol. Sci..

[16] Young I.C., Benhabbour S.R. (2021). Multipurpose prevention technologies: Oral, parenteral, and vaginal dosage forms for prevention of HIV/STIs and unplanned pregnancy. Polymers.

[17] Tian Y. (2024). Check for updates Chapter 22 Polyvinylamine and Its Derivative as Effective Carrier for Targeted Delivery of Small RNAs. RNA Amplif. Anal. Methods Protoc..

[18] Wang Y., Jia Z., Jiang J., Mao X., Pan X., Wu J. (2019). Highly regioselective ring-opening polymerization of cyclic diester for alternating sequence-controlled copolymer synthesis of mandelic acid and glycolic acid. Macromolecules.

[19] Molotkovsky R.J., Alexandrova V.V., Galimzyanov T.R., Jiménez-Munguía I., Pavlov K.V., Batishchev O.V., Akimov S.A. (2018). Lateral membrane heterogeneity regulates viral-induced membrane fusion during HIV entry. Int. J. Mol. Sci..

[20] Chang Y., Xu H., Motaleb M.A., Liu J. (2021). Characterization of the flagellar collar reveals structural plasticity essential for spirochete motility. mBio.

[21] Ghunawat S., Sarkar R., Garg V.K. (2019). Comparative Study of 35% Glycolic Acid, 20% Salicylic–10% Mandelic Acid, and Phytic Acid Combination Peels in the Treatment of Active Acne and Postacne Pigmentation. J. Cutan. Aesthetic Surg..

[22] Edison B.L., Smith H.A., Li W.H., Parsa R., Green B.A., Konish P., Dufort M., Tierney N.K. (2020). 18295 Mandelic Acid, a Lipophilic Alpha Hydroxy Acid, Reduces Lipid Production, Enhances Exfoliation and Provides Clinical and Patient Perceivable Benefits to Oily and Photodamaged Skin. J. Am. Acad. Dermatol..

[23] Müller E., Sosedov O., Gröning J.A.D., Stolz A. (2021). Synthesis of (R)-mandelic acid and (R)-mandelic acid amide by recombinant *E. coli* strains expressing a (R)-specific oxynitrilase and an arylacetonitrilase. Biotechnol. Lett..

[24] Jacobs S.W., Culbertson E.J. (2018). Effects of Topical Mandelic Acid Treatment on Facial Skin Viscoelasticity. Facial Plast. Surg..

[25] Saeed A., Shahzad D., Faisal M., Larik F.A., El-Seedi H.R., Channar P.A. (2017). Developments in the synthesis of the antiplatelet and antithrombotic drug (S)-clopidogrel. Chirality.

[26] Bhalla T.C., Thakur N., Kumar V. (2024). Arylacetonitrilases: Potential Biocatalysts for Green Chemistry. Appl. Biochem. Biotechnol..

[27] Masumoto S., Suzuki M., Kanai M., Shibasaki M. (2002). A practical synthesis of (S)-oxybutynin. Tetrahedron Lett..

[28] Dash R., Biswal J., Yadav M., Sharma T., Mohapatra S., Prusty S.K. (2023). Novel atorvastatin-curcumin conjugate nanogel, a selective COX2 inhibitor with enhanced biopharmaceutical profile: Design, synthesis, in silico, in vitro, and in vivo investigation. J. Drug Deliv. Sci. Technol..

[29] Lin D., Xu X., Chen L., Chen L., Deng M., Chen J., Ren Z., Lei L., Wang J., Deng J. (2023). Supramolecular nanofiber of indomethacin derivative confers highly cyclooxygenase-2 (COX-2) selectivity and boosts anti-inflammatory efficacy. J. Control. Release.

[30] Cao S., Zhao B., Zhang X., Hou X. (2024). Advances in the application of photocatalysis, electrocatalysis, and biocatalysis in the synthesis of mandelic acid. Shandong Chem. Ind..

[31] Zhang Y., Su C., Lei J., Chen L., Hu H., Zeng S., Yu L. (2019). Studies on the L-2-hydroxy-acid oxidase 2 catalyzed metabolism of S-mandelic acid and its analogues. Drug Metab. Pharmacokinet..

[32] Dai Y., Wang J., Tao Z., Luo L., Huang C., Liu B., Shi H., Tang L., Ou Z. (2024). Highly efficient synthesis of the chiral ACE inhibitor intermediate (R)-2-hydroxy-4-phenylbutyrate ethyl ester via engineered bi-enzyme coupled systems. Bioresour. Bioprocess..

[33] Lu Y., Swisher J.H., Meyer T.Y., Coates G.W. (2021). Chirality-directed regioselectivity: An approach for the synthesis of alternating poly (lactic-co-glycolic acid). J. Am. Chem. Soc..

[34] Carvalho P.D.S., Tenorio J.C., Alves D.C. (2024). Diastereomeric Double Salts of Carvedilol with Mandelic Acids. A Structural Analysis Reveals the Failure in Resolution. SSRN.

[35] Belcher R., Sykes A., Tatlow J.C. (1954). Mandelic acid and halogen-substituted mandelic acids as reagents for the determination of zirconium. Anal. Chim. Acta.

[36] Zambare A.A., Bagal M.S., Sharma S.J., Sekar M. (2024). NLOphoric unsymmetrically substituted D-π-A benzodifuranone dyes: Density functional theory, time dependent-density functional theory, and non-linear optical studies. Comput. Theor. Chem..

[37] Jeswani H.K., Perry M.R., Shaver M.P., Azapagic A. (2023). Biodegradable and conventional plastic packaging: Comparison of life cycle environmental impacts of poly (mandelic acid) and polystyrene. Sci. Total Environ..

[38] Pavlačková J., Egner P., Mokrejš P., Janalíková M. (2024). Formulating Sustainable Emulsions: Mandelic Acid and Essential Oils as Natural Preservatives. Molecules.

[39] Qu T., Shao Y., Csinos A.S., Ji P. (2016). Sensitivity of Phytophthora nicotianae From Tobacco to Fluopicolide, Mandipropamid, and Oxathiapiprolin. Plant Disaster.

[40] Li S., Li D., Xiao T., Zhang S., Song Z., Ma H. (2016). Design, Synthesis, Fungicidal Activity, and Unexpected Docking Model of the First Chiral Boscalid Analogues Containing Oxazolines. J. Agric. Food Chem..

[41] Hou S., Xie D., Yang J., Niu X., Hu D., Wu Z. (2021). Design, synthesis and antifungal evaluation of novel mandelic acid derivatives containing a 1,3,4-oxadiazothioether moiety. Chem. Biol. Drug Des..

[42] Hao K., Lin B., Nian F., Gao X., Wei Z., Luo G., Lu Y., Lan M., Yang J., Wu G. (2019). RNA-seq analysis of the response of plant-pathogenic oomycete *Phytophthora parasitica* to the fungicide dimethomorph. Rev. Argent. Microbiol..

[43] Wang J., Shi H., Lu A. (2024). Design, Synthesis, and Antifungal/Anti-Oomycete Activities of Novel 1, 2, 4-Triazole Derivatives Containing Carboxamide Fragments. J. Fungi.

[44] Zheng Y., Shi D., Song D., Chen K., Wen F., Zhang J., Xue W., Wu Z. (2024). Novel mandelic acid derivatives containing piperazinyls as potential candidate fungicides against *Monilinia* fructicola: Design, synthesis and mechanism study. Bioorganic Chem..

[45] Peng Y., Chen B. (2024). Role of cell membrane homeostasis in the pathogenicity of pathogenic filamentous fungi. Virulence.

[46] Guan X. (2013). Functional Analysis of Cytoskeletal Signalling in the Defence Response of Grapevine. Ph.D. Thesis.

[47] Liu C., Wu M., Qu J., Huang X., Zeng Q., Ha M. (2022). JNK and Jag1/Notch2 co-regulate CXCL16 to facilitate cypermethrin-induced kidney damage. Ecotoxicol. Environ. Saf..

[48] Bera P., Biswas S. (2024). Nanoscale Zr(IV) MOF-Embedded Chitosan on Paper Composite for Aqueous Phase Detection of the Herbicide Metamitron and the Food Colorant Tartrazine. ACS Appl. Nano Mater..

[49] Huang Q., Tao Y., Li H., Guo L., Wang L., Ban C., Shen G. (2021). (R,S)-Mandelic acid in pure and binary solvents solubility measurement and its correlation with thermodynamic models. J. Mol. Liq..

[50] Nandanwar S.U., Rathod S., Bansal V., Bokade V.V. (2021). A Review on Selective Production of Acetophenone from Oxidation of Ethylbenzene over Heterogeneous Catalysts in a Decade. Catal. Lett..

[51] Henry M.C., Minty L., Kwok A.C.W., Elwood J.M.L., Foulis A.J., Pettinger J., Jamieson C. (2024). One-Pot Oxidative Amidation of Aldehydes via the Generation of Nitrile Imine Intermediates. J. Org. Chem..

[52] Yadav G.D., Sowbna P.R. (2012). Process intensification and waste minimization in liquid–liquid–liquid phase transfer catalyzed selective synthesis of mandelic acid. Chem. Eng. Res. Des..

[53] Battaglia V., Meninno S., Lattanzi A. (2025). Asymmetric Organocatalysed Synthesis of (R)-Mandelic Acid Esters and α-Alkoxy Derivatives from Commercial Sources. Chem. Eur. J..

[54] Mao Z., Xia K., Zhang K., Chen H., Li M., Abdukader A., Jin W. (2024). Visible light-induced oxidative esterification of mandelic acid with alcohols: A new synthesis of α-ketoesters. Green Chem..

[55] Xia Y., Zhao J., Saeed M., Hussain N., Chen X., Guo Z., Yong Y., Chen H. (2024). Molecular modification strategies of nitrilase for its potential application in agriculture. J. Agric. Food Chem..

[56] Shen J.D., Cai X., Liu Z.Q., Zheng Y.G. (2021). Nitrilase: A promising biocatalyst in industrial applications for green chemistry. Crit. Rev. Biotechnol..

[57] Liu T.T., Su W.C., Chen Q.X., Shen D.Y., Zhuang J.X. (2020). The inhibitory kinetics and mechanism of glycolic acid on lipase. J. Biomol. Struct. Dyn..

[58] Kobayashi T., Nakajima-Kambe T. (2024). Isolation and characterization of a novel bacterium that promotes the degradation of poly (glycolic acid) by its extracellular esterase under thermophilic conditions. Polym. Degrad. Stab..

[59] Sulej J., Piątek-Gołda W., Grąz M., Szałapata K., Waśko P., Janik-Zabrotowicz E., Osińska-Jaroszuk M. (2023). Immobilisation of cellobiose dehydrogenase and laccase on chitosan particles as a multi-enzymatic system for the synthesis of lactobionic acid. J. Funct. Biomater..

[60] Wang P., Li D., Yang J., Jiang L., Feng J., Yang C., Shi R. (2014). Immobilization of (S)-mandelate dehydrogenase and its catalytic performance on stereoselective transformation of mandelic acid. J. Taiwan Inst. Chem. Eng..

[61] Stolz A., Eppinger E., Sosedov O., Kiziak C. (2019). Comparative Analysis of the Conversion of Mandelonitrile and 2-Phenylpropionitrile by a Large Set of Variants Generated from a Nitrilase Originating from *Pseudomonas fluorescens* EBC191. Molecules.

[62] Sun H., Wang H., Gao W., Chen L., Wu K., Wei D. (2015). Directed evolution of nitrilase PpL19 from *Pseudomonas psychrotolerans* L19 and identification of enantio-complementary mutants toward mandelonitrile. Biochem. Biophys. Res. Commun..

[63] Bian S.Q., Wang Z.K., Gong J.S., Su C., Li H., Xu Z.H., Shi J.S. (2025). Protein Engineering of Substrate Specificity toward Nitrilases: Strategies and Challenges. J. Agric. Food Chem..

[64] Baum S., Williamson Dael S., Sewell T., Stolz A. (2012). Conversion of Sterically Demanding α,α-Disubstituted Phenylacetonitriles by the Arylacetonitrilase from *Pseudomonas fluorescens* EBC191. Appl. Environ. Microbiol..

[65] Zhang X.H., Liu Z.Q., Xue Y.P., Wang Y.S., Yang B., Zheng Y.G. (2018). Production of R-Mandelic Acid Using Nitrilase from Recombinant *E. coli* Cells Immobilized with Tris (Hydroxymethyl) Phosphine. Appl. Biochem. Biotechnol..

[66] Arana-Peña S., Rios N.S., Carballares D., Gonçalves L.R.B., Fernandez-Lafuente R. (2021). Immobilization of lipases via interfacial activation on hydrophobic supports: Production of biocatalysts libraries by altering the immobilization conditions. Catal. Today.

[67] Lima R.N., Anjos C.S., Porto A.L. (2022). Biocatalytic synthesis of lipophilic amides by the lipase of *Candida antarctica* type B. Mol. Catal..

[68] Chen X., Yang C., Wang P., Zhang X., Bao B., Li D., Shi R. (2017). Stereoselective biotransformation of racemic mandelic acid using immobilized laccase and (S)-mandelate dehydrogenase. Bioresour. Bioprocess..

[69] Arregui L., Ayala M., Gómez-Gil X., Gutiérrez-Soto G., Hernández-Luna C.E., Santos M.H., Levin L., Rojo-Domínguez A., Romero-Martínez D., Saparrat M.C.N. (2019). Laccases: Structure, function, and potential application in water bioremediation. Microb. Cell Factories.

[70] Elgahwash R.G.A., Blažić M., Balaž A.M., Prodanović R. (2024). Lactobionic acid production via mutant cellobiose dehydrogenase/laccase continuous enzymatic regeneration of electron acceptors. Biocatal. Biotransform..

[71] Luo Z., Wu G., Kong M., Chen Z., Zhuang Z., Fan J., Chen T. (2023). Structured illumination-based super-resolution live-cell quantitative FRET imaging. Photonics Res..

[72] Reifenrath M., Boles E. (2018). Engineering of hydroxymandelate synthases and the aromatic amino acid pathway enables de novo biosynthesis of mandelic and 4-hydroxymandelic acid with *Saccharomyces cerevisiae*. Metab. Eng..

[73] Lukito B.R., Wang Z., Balaji S.S., Li Z. (2021). Production of (R)-mandelic acid from styrene, L-phenylalanine, glycerol, or glucose via cascade biotransformations. Bioresour. Bioprocess..

[74] Lv B., Zeng Y., Zhang H., Li Z., Xu Z., Wang Y., Gao Y., Chen Y., Fu X. (2022). Mechanosensitive channels mediate hypoionic shock-induced aminoglycoside potentiation against bacterial persisters by enhancing antibiotic uptake. Antimicrob. Agents Chemother..

[75] Nie Z., Liu P., Wang Y., Guo X., Tan Z., Shen J., Tang Z., Lin J., Sun J., Zheng P. (2021). Directed evolution and rational design of mechanosensitive channel MscCG2 for improved glutamate excretion efficiency. J. Agric. Food Chem..

[76] Balleza D., Alessandrini A., Beltrán García M.J. (2019). Role of lipid composition, physicochemical interactions, and membrane mechanics in the molecular actions of microbial cyclic lipopeptides. J. Membr. Biol..

[77] Kawasaki H., Martinac B. (2020). Mechanosensitive channels of Corynebacterium glutamicum functioning as exporters of l-glutamate and other valuable metabolites. Curr. Opin. Chem. Biol..

[78] Mateo C., Chmura A., Rustler S., Rantwijk F., Stolz A., Sheldon R.A. (2006). Synthesis of enantiomerically pure (S)-mandelic acid using an oxynitrilase–nitrilase bienzymatic cascade: A nitrilase surprisingly shows nitrile hydratase activity. Tetrahedron Asymmetry.

[79] Rustler S., Motejadded H., Altenbuchner J., Stolz A. (2008). Simultaneous expression of an arylacetonitrilase from *Pseudomonas fluorescens* and a (S)-oxynitrilase from Manihot esculenta in Pichia pastoris for the synthesis of (S)-mandelic acid. Appl. Microbiol. Biotechnol..

[80] Chmura A., Rustler S., Paravidino M., Rantwijk F., Stolz A., Sheldon R.A. (2013). The combi-CLEA approach: Enzymatic cascade synthesis of enantiomerically pure (S)-mandelic acid. Tetrahedron Asymmetry.

[81] Rucká L., Volkova O., Pavlík A., Kaplan O., Kracík M., Nešvera J., Martínková L., Pátek M. (2014). Expression control of nitrile hydratase and amidase genes in *Rhodococcus erythropolis* and substrate specificities of the enzymes. Antonie Leeuwenhoek.

[82] Zhou S.P., Xue Y.P., Zheng Y.G. (2024). Maximizing the potential of nitrilase: Unveiling their diversity, catalytic proficiency, and versatile applications. Biotechnol. Adv..

[83] Dadashipour M., Asano Y. (2011). Hydroxynitrile lyases: Insights into biochemistry, discovery, and engineering. ACS Catal..

[84] Sheldon R.A., van Pelt S. (2013). Enzyme immobilisation in biocatalysis: Why, what and how. Chem. Soc. Rev..

[85] Chen S., Liu F., Zhang K., Huang H., Wang H., Zhou J., Zhang J., Gong Y., Zhang D., Chen Y. (2016). An efficient enzymatic aminolysis for kinetic resolution of aromatic α-hydroxyl acid in non-aqueous media. Tetrahedron Lett..

[86] He Y.C., Ma C.L., Zhang X., Li L., Xu J.H., Wu M.X. (2013). Highly enantioselective oxidation of racemic phenyl-1,2-ethanediol to optically pure (R)-(-)-mandelic acid by a newly isolated *Brevibacterium lutescens* CCZU12-1. Appl. Microbiol. Biotechnol..

[87] Gennaro P.D., Bernasconi S., Orsini F., Corretto E., Sello G. (2010). Multienzymatic preparation of 3-[(1R)-1-hydroxyethyl]benzoic acid and (2S)-hydroxy(phenyl)ethanoic acid. Tetrahedron Asymmetry.

[88] Sun Z., Ning Y., Liu L., Liu Y., Sun B., Jiang W., Yang C., Yang S. (2011). Metabolic engineering of the L-phenylalanine pathway in *Escherichia coli* for the production of S- or R-mandelic acid. Microb. Cell Factories.

[89] Huang H., Xu J. (2006). Preparation of (S)-mandelic acid from racemate using growing cells of *Pseudomonas putida* ECU1009 with (R)-mandelate degradation activity. Biochem. Eng. J..

[90] Takakura Y., Ono T., Danjo K., Nozaki H. (2022). Efficient enzymatic production of benzaldehyde from l-phenylalanine with a mutant form of 4-hydroxymandelate synthase. Biosci. Biotechnol. Biochem..

[91] Brownlee J., He P., Moran G.R., Harrison D.H.T. (2008). Two Roads Diverged:  The Structure of Hydroxymandelate Synthase from *Amycolatopsis orientalis* in Complex with 4-Hydroxymandelate. Biochemistry.

[92] Luo Z., Lee S. (2020). Metabolic engineering of *Escherichia coli* for the production of benzoic acid from glucose. Metab. Eng..

[93] Lukito B.R., Sekar B.S., Wu S., Li Z. (2019). Whole Cell-Based Cascade Biotransformation for the Production of (S)-Mandelic Acid from Styrene, L-Phenylalanine, Glucose, or Glycerol. Adv. Synth. Catal..

[94] Yao C., Cao Y., Wu S., Li S., He B. (2013). An organic solvent and thermally stable lipase from *Burkholderia ambifaria* YCJ01: Purification, characteristics and application for chiral resolution of mandelic acid. J. Mol. Catal. B Enzym..

[95] Cao Y., Wu S., Li J., Wu B., He B. (2014). Highly efficient resolution of mandelic acid using lipase from *Pseudomonas stutzeri* LC2-8 and a molecular modeling approach to rationalize its enantioselectivity. J. Mol. Catal. B Enzym..

[96] Guo F., Ye L., Li A., Yang X., Yang C., Yu H. (2016). Insight into the role of halogen bond in the activity of d-mandelate dehydrogenase toward halogenated substrates. Tetrahedron Lett..

[97] Durao P., Bento I., Fernandes A.T., Melo E.P., Lindley P.F., Martins L.O. (2006). Perturbations of the T1 copper site in the CotA laccase from *Bacillus subtilis*: Structural, biochemical, enzymatic and stability studies. JBIC J. Biol. Inorg. Chem..

[98] Hitaishi V.P., Clément R., Quattrocchi L., Parent P., Duché D., Zuily L., Ilbert M., Lojou E., Mazurenko L. (2019). Interplay between orientation at electrodes and copper activation of Thermus thermophilus laccase for O_2_ reduction. J. Am. Chem. Soc..

[99] Durao P., Chen Z., Silva C.S., Soares C.M., Pereira M.M., Todorovic S., Hildebrandt P., Bento I., Lindley P.F., Martins L.O. (2008). Proximal mutations at the type 1 copper site of CotA laccase: Spectroscopic, redox, kinetic and structural characterization of I494A and L386A mutants. Biochem. J..

[100] Janusz G., Pawlik A., Świderska-Burek U., Polak J., Sulej J., Jarosz-Wilkołazka A., Paszczyński A. (2020). Laccase properties, physiological functions, and evolution. Int. J. Mol. Sci..

[101] Dewanti A.R., Mitra B.A. (2003). Transient Intermediate in the Reaction Catalyzed by (S)-Mandelate Dehydrogenase from *Pseudomonas putida*. Biochemistry.

[102] Blank L.M., Ebert B.E., Buehler K., Bühler B. (2010). Redox Biocatalysis and Metabolism: Molecular Mechanisms and Metabolic Network Analysis. Antioxid. Redox Signal..

[103] Bell E.L., Finnigan W., France S.P., Green A.P., Hayes M.A., Hepworth L.J., Lovelock S.L., Niikura H., Osuna S., Romero E. (2021). Biocatalysis. Nat. Rev. Methods Primers.

[104] Wang Z., Balaji S., Sekar Li Z. (2021). Recent advances in artificial enzyme cascades for the production of value-added chemicals. Bioresour. Technol..

[105] Gong X., Zhang J., Gan Q., Teng Y., Hou J., Lyu Y., Liu Z., Wu Z., Dai R., Zou Y. (2024). Advancing microbial production through artificial intelligence-aided biology. Biotechnol. Adv..

[106] Sekar B.S., Lukito B.R., Li Z. (2019). Production of Natural 2-Phenylethanol from Glucose or Glycerol with Coupled *Escherichia coli* Strains Expressing l-Phenylalanine Biosynthesis Pathway and Artificial Biocascades. ACS Sustain. Chem. Eng..

[107] Xu Y., Wu Y., Lv X., Sun G., Zhang H., Chen T., Du G., Li J., Liu L. (2021). Design and construction of novel biocatalyst for bioprocessing: Recent advances and future outlook. Bioresour. Technol..

[108] Xue C., Liu F., Xu M., Tang I.C., Zhao J., Bai F., Yang S.T. (2016). Butanol production in acetone-butanol-ethanol fermentation with in situ product recovery by adsorption. Bioresour. Technol..

[109] Lukito B.R., Wu S., Saw H.J.J., Li Z. (2019). One-Pot Production of Natural 2-Phenylethanol from L-Phenylalanine via Cascade Biotransformations. ChemCatChem.

[110] Zhou Y., Sekar B.S., Wu S., Li Z. (2020). Benzoic acid production via cascade biotransformation and coupled fermentation-biotransformation. Biotechnol. Bioeng..

[111] Zhou L., Tao C., Shen X., Sun X., Wang J., Yuan Q. (2024). Unlocking the potential of enzyme engineering via rational computational design strategies. Biotechnol. Adv..

[112] Wang H., Liu R., Wang B., Hong Y., Cui Z., Ni Q. (2023). Multitype Perception Method for Drug-Target Interaction Prediction. IEEE/ACM Trans. Comput. Biol. Bioinform..

[113] Casilli F., Canyelles-Niño M., Roelfes G., Alonso-Cotchico L. (2024). Computation-guided engineering of distal mutations in an artificial enzyme. Faraday Discuss..

[114] Goshisht M.K. (2024). Machine Learning and Deep Learning in Synthetic Biology: Key Architectures, Applications, and Challenges. ACS Omega.

[115] Volk M.J., Lourentzou I., Mishra S., Vo L.T., Zhai C., Zhao H. (2020). Biosystems Design by Machine Learning. ACS Synth. Biol..

